# Self-Explaining Neural Networks for Transparent Parkinson’s Disease Screening

**DOI:** 10.3390/s26092671

**Published:** 2026-04-25

**Authors:** Mahmoud E. Farfoura, Ahmad A. A. Alkhatib, Tee Connie

**Affiliations:** 1 Cybersecurity Department, Al-Zaytoonah University of Jordan, Amman 11733, Jordan; 2 Faculty of Information Science & Technology, Multimedia University, Jalan Ayer Keroh Lama, Melaka 75450, Malaysia

**Keywords:** Parkinson’s disease detection, self-explaining neural networks, explainable artificial intelligence, gait analysis, ground reaction force, intrinsic interpretability, residual CNN, concept learning, wearable sensors, clinical decision support

## Abstract

Transparent clinical decision-making remains a critical barrier to deploying deep learning in medical diagnosis. Post hoc explanation methods approximate model behaviour after training but cannot guarantee that explanations faithfully reflect the underlying reasoning. This study proposes a Self-Explaining Neural Network (SENN) for Parkinson’s Disease (PD) screening via Ground Reaction Force (GRF) gait analysis, enforcing intrinsic interpretability through learnable basis concepts and input-dependent relevance scores computed jointly with the prediction. The architecture combines a four-block residual CNN backbone with stochastic depth regularisation, a 16-concept encoder with diversity and stability constraints, and temperature-scaled probability calibration for reliable clinical operating points. Evaluated on the PhysioNet Gait in Parkinson’s Disease dataset (306 subjects, 16 GRF sensors per foot), SENN achieves a subject-level ROC-AUC of 0.916 [95% CI: 0.867–0.964], sensitivity of 0.913 [0.862–0.963], specificity of 0.671 [0.485–0.858], and Average Precision of 0.942 [0.918–0.967], reported across five independent random seeds. Comparative evaluation against four deep learning baselines—CNN-Residual, BiLSTM, CNN-LSTM, and CNN-Attention—confirms that the interpretability constraints impose no statistically significant reduction in discriminative performance, with all pairwise ROC-AUC confidence intervals overlapping. Concept-level analysis reveals that the three most discriminative concepts correspond to disrupted midfoot loading patterns, increased step-length variability, and bilateral cadence asymmetry—all established biomechanical hallmarks of parkinsonian gait—providing clinically grounded, patient-specific explanations without post hoc approximation. These findings demonstrate that rigorous intrinsic interpretability and competitive predictive accuracy are simultaneously achievable in deep gait analysis, supporting the clinical adoption of transparent diagnostic AI.

## 1. Introduction

Parkinson’s disease (PD) continues to pose a substantial and growing global health burden, underscoring the need to move beyond reliance on purely clinical observation toward a more biologically informed and objective diagnostic framework. Although traditional diagnostic systems emphasise motor symptoms such as bradykinesia, rigidity, and postural instability, emerging evidence shows that these subjective assessments are often insufficient—particularly in the early or prodromal phases, where symptoms may be subtle or atypical. Recent guidelines highlight that PD encompasses a broader spectrum of etiologies than previously recognised and emphasise the importance of integrating clinical evaluation with imaging, genetic testing, and fluid biomarkers to strengthen diagnostic accuracy. These updated recommendations stress that objective biological measures, alongside long-term clinical follow-up, are essential for identifying PD earlier and improving the precision of disease characterisation and management [1].

In the search for such objective markers, the analysis of gait via ground reaction force (GRF) sensors has proven to be highly sensitive to dopaminergic deficits. Instrumented insoles positioned beneath each foot record the vertical force exerted during walking at 100 Hz, capturing subtle disruptions in heel strike, midfoot loading, and toe-off dynamics that are characteristic of Parkinsonian locomotion. The high dimensionality and temporal complexity of multi-sensor GRF time-series data has made Deep Learning (DL) the dominant analytical approach in this domain. A comprehensive review by Skaramagkas et al. confirms that DL models significantly outperform traditional statistical methods [2] in decoding these gait patterns, effectively handling the non-linearity inherent in force sensor data [3]. Consequently, automated gait analysis is increasingly viewed as a viable surrogate for expert clinical assessment.

However, the clinical translation of these high-performance DL models is stalled by their inherent lack of transparency, often referred to as the “black box” problem. In healthcare ecosystems, diagnostic accuracy cannot come at the expense of interpretability; clinicians require a clear understanding of the decision logic to validate automated outputs against their medical training. Athamneh et al. emphasise that Explainable Artificial Intelligence (XAI) is no longer optional but essential, arguing that without mechanisms to trace predictions back to tangible physiological features, AI tools cannot meet the safety and trust requirements of modern medicine [4]. Critically, most post hoc explanation methods—including SHAP, LIME, and gradient-based saliency maps—approximate model behaviour after training and therefore cannot guarantee that their explanations faithfully reflect the underlying reasoning process.

To address the tension between accuracy and interpretability, this work proposes a Self-Explaining Neural Network (SENN) that enforces intrinsic interpretability by design rather than approximating it after the fact. Following the SENN formulation of Alvarez-Melis and Jaakkola [5], the model decomposes raw GRF inputs into a set of smooth, human-aligned gait concepts and assigns input-dependent relevance scores that explain how each concept influences the final decision for each individual patient. This structure yields a concept-based reasoning process without sacrificing classification performance, providing clinically grounded justification that is biologically anchored in established PD gait biomarkers such as step-length variability, bilateral cadence asymmetry, and midfoot loading disruption.

Despite the growing adoption of deep learning for PD detection, the simultaneous achievement of competitive predictive accuracy and genuine, intrinsic interpretability on GRF gait data remains an open challenge. This work addresses this limitation through the following contributions:

1. **Interpretable-by-Design Architecture:** We propose a SENN framework built on a four-block residual CNN backbone with stochastic depth regularisation, a 16-concept encoder with diversity and stability constraints, and temperature-scaled probability calibration. Unlike post hoc XAI methods, the model’s explanations are structurally guaranteed to reflect its actual decision process.
2. **Biologically grounded concept discovery:** The learned concepts are systematically linked to established PD gait biomarkers through sensor-correlation analysis. Dominant concepts are shown to correspond to disrupted midfoot loading patterns, increased step-length variability, bilateral cadence asymmetry, and reduced toe-off impulse—providing clinicians with a physiologically interpretable pathway from raw sensor data to diagnosis.
3. **Rigorous statistical validation:** The framework is evaluated with subject-level holdout (306 subjects, 92 test), five independent random seeds, and 95% confidence intervals computed via t-distribution (df = 4). This methodology addresses the window-level evaluation inflations prevalent in the prior literature and produces reliable performance estimates: ROC-AUC of 0.916 [0.867–0.964], sensitivity of 0.913 [0.862–0.963], and Average Precision of 0.942 [0.918–0.967].
4. **Interpretability without accuracy cost:** Comparative evaluation against four deep learning baselines—CNN-Residual, BiLSTM, CNN-LSTM, and CNN-Attention—demonstrates that the interpretability constraints impose no statistically significant reduction in discriminative performance, with all pairwise ROC-AUC confidence intervals overlapping. This establishes that rigorous intrinsic interpretability and competitive predictive accuracy are simultaneously achievable in deep GRF gait analysis.

The paper is organised as follows: Section 2 reviews related work on deep learning for PD gait analysis and interpretable AI in clinical settings. Section 3 details the SENN architecture, training procedure, and calibration methodology. Section 4 presents the experimental dataset, evaluation protocol, and results including the biological concept interpretation. Section 5 concludes with a summary of findings and directions for future work.

## 2. Related Works

### 2.1. Wearable Sensor-Based Gait Analysis

The objective quantification of gait has traditionally relied on wearable sensors, particularly force-sensitive insoles and inertial measurement units, due to their high temporal resolution and clinical deployability. Ground reaction force (GRF) sensors embedded beneath the foot capture the vertical force profile throughout the gait cycle at 100 Hz, encoding biomechanically rich information about heel strike, midfoot loading, and toe-off dynamics. Huang et al. conducted a systematic review demonstrating that sensor-based systems achieve high precision in measuring stride time variability and freezing of gait episodes [6]. While effective, these systems often require careful calibration and structured walking protocols that can limit scalability in unsupervised clinical settings. To mitigate this, Wang et al. proposed a deep learning framework fusing data from multiple body-worn sensors, achieving robust classification accuracy across gait conditions [7]. However, the cost and complexity of synchronised multi-sensor arrays remain significant barriers to widespread adoption. Khare et al. further noted that while 1D sensor signals can be processed efficiently with convolutional architectures, the temporal variability and subject-specific noise in GRF recordings demand careful segmentation and normalisation strategies to avoid information leakage between training and test subjects [8].

### 2.2. Vision-Based Motion Capture

To overcome the physical burden of wearable sensors, a parallel line of research has pursued vision-based markerless motion capture for gait analysis. Sato et al. validated the efficacy of single-camera systems to quantify turning behaviour, confirming that vision-based metrics correlate strongly with clinical UPDRS scores [9]. Similarly, Ripic et al. developed a non-contact screening system using RGB cameras, demonstrating that computer vision can effectively extract gait parameters without physical markers [10]. While promising, these systems are highly sensitive to environmental factors such as lighting and occlusion. Rupprechter et al. addressed accessibility by utilising smartphone cameras for remote monitoring [11]. Broom et al. applied skeletal transformation techniques to 3D pose data, yet their approach focused on raw topology rather than identifying which specific skeletal segments drove the diagnostic decision [12]. In contrast, the present work operates on GRF sensor data—a modality that is both environmentally robust and directly coupled to the biomechanical forces that characterise Parkinsonian locomotion, without requiring line-of-sight or controlled illumination.

### 2.3. Graph and Recurrent Neural Architectures

Given the sequential and topological nature of gait, advanced deep learning architectures have become standard for modelling kinematic data. Yin et al. introduced a Spatial–Temporal Joint Attention Graph Convolutional Network (STJA-GCN) that models human joint connectivity using a refined skeleton graph and multi-branch spatial–temporal attention, achieving substantial improvements in abnormal gait recognition accuracy [13]. Tran et al. proposed a Multi-Model Long Short-Term Memory (LSTM) network for gait recognition using window-based data segments, demonstrating that recurrent architectures effectively capture temporal dynamics in sequential gait recordings [14]. Zeng et al. proposed a video-based Skeleton-Silhouette Fusion Convolutional Network that combines skeletal and silhouette representations to enhance gait assessment in movement disorders [15]. Tao et al. presented a gait phase detection method integrating an LSTM with a Hidden Markov Model using foot-mounted inertial sensors, achieving high phase classification accuracy [16]. Despite their discriminative power, all of these architectures operate as opaque black boxes, offering no mechanistic insight into which biomechanical features drove a specific prediction—a critical limitation for clinical deployment where individual predictions must be auditable.

### 2.4. Multimodal Fusion Strategies

A growing body of work has sought to improve diagnostic accuracy by fusing heterogeneous data modalities. Bensefia et al. introduced a CNN-based framework for offline handwriting analysis in PD detection, demonstrating that deep convolutional features extracted from static handwriting samples can effectively discriminate between PD patients and healthy controls without requiring dynamic pen-kinematics data [17]. Khedimi et al. presented a deep learning ensemble framework for PD detection and motor severity prediction based on voice analysis, achieving high accuracy across multiple evaluation conditions [18]. Rangel-Cascajosa et al. proposed a gait-based detection method using recurrent neural networks tailored for wearable systems, demonstrating strong performance in real-time applicability [19]. However, fusion models introduce an additional interpretability challenge: the black-box fusion layers obscure which modality—voice, handwriting, or gait—drove any individual diagnosis. Furthermore, the logistical requirement for synchronised multimodal data collection constrains the clinical utility of these approaches relative to single-modality GRF-based pipelines, which can be deployed using a single pair of instrumented insoles during a standard two-minute walking assessment.

### 2.5. Post Hoc Explainability Limitations

The opacity of deep learning has motivated substantial interest in post hoc explainability methods, particularly SHAP and LIME. Nayan et al. presented an interpretable machine learning framework for PD prediction incorporating feature engineering and XAI techniques to enhance decision transparency [20]. Esan et al. explored interpretable machine learning models for PD prediction, demonstrating that gradient-based attribution methods can highlight feature importance while maintaining competitive accuracy [21]. Shoeibi et al. presented an OCT-based explainable AI framework using SHAP and LIME to improve transparency in PD diagnosis [22]. Despite their utility, post hoc methods suffer from a fundamental limitation: they approximate explanation surfaces fitted to model outputs after training, and therefore cannot guarantee that the explanations faithfully represent the model’s internal reasoning. Instability across runs, sensitivity to hyperparameter choices, and the inability to enforce biological plausibility remain unresolved. This highlights a structural limitation of the post hoc paradigm: such methods explain where a model attended in the input space, but not what biomechanical concept it identified or how that concept was weighted in the final decision.

### 2.6. The Shift to Interpretable-by-Design Models

Acknowledging the pitfalls of post hoc explanations, the field is moving toward architectures that embed interpretability as a structural constraint rather than a post-processing step. Barbiero et al. introduced entropy-based logic layers to extract explicit rules from neural networks [23], though this approach has yet to be successfully validated on complex temporal force sensor data. Ciravegna et al. proposed Logic Explained Networks (LENs) to enforce parsimony in explanations, but reported a significant accuracy trade-off relative to unconstrained deep learning [24]. In the image domain, Chen et al. developed ProtoPNet, which classifies images based on similarity to learned prototypes [25]—a compelling paradigm that is, however, ill-suited for dynamic time-series modalities such as GRF signals, where meaningful “prototypes” are temporal patterns rather than spatial patches.

The literature reveals a persistent dichotomy: high-performance models are opaque, while interpretable models either sacrifice accuracy or cannot accommodate the temporal complexity of GRF data. This work bridges this gap by proposing a Self-Explaining Neural Network (SENN) for PD screening from multi-sensor GRF recordings. Unlike post hoc methods that approximate decision boundaries after training [20,21,22], our SENN architecture incorporates a 16-concept encoder directly into the forward pass, jointly learning basis concepts and their input-dependent relevance scores end-to-end. This design enforces the learning of distinct, biologically grounded gait concepts—functionally analogous to the prototypes of Chen et al. [25] but adapted for temporal force dynamics—without the accuracy trade-off observed in logic-based networks [23,24]. Crucially, concepts are regularised for diversity, stability, and sparsity, ensuring that the learned representations correspond to independent biomechanical phenomena rather than redundant activations. Evaluated across five independent random seeds with subject-stratified splits and 95% confidence intervals, the proposed framework achieves competitive performance against four deep learning baselines while providing patient-specific, biologically interpretable explanations grounded in established PD gait biomarkers.

## 3. Materials and Methods

**Figure 1:**
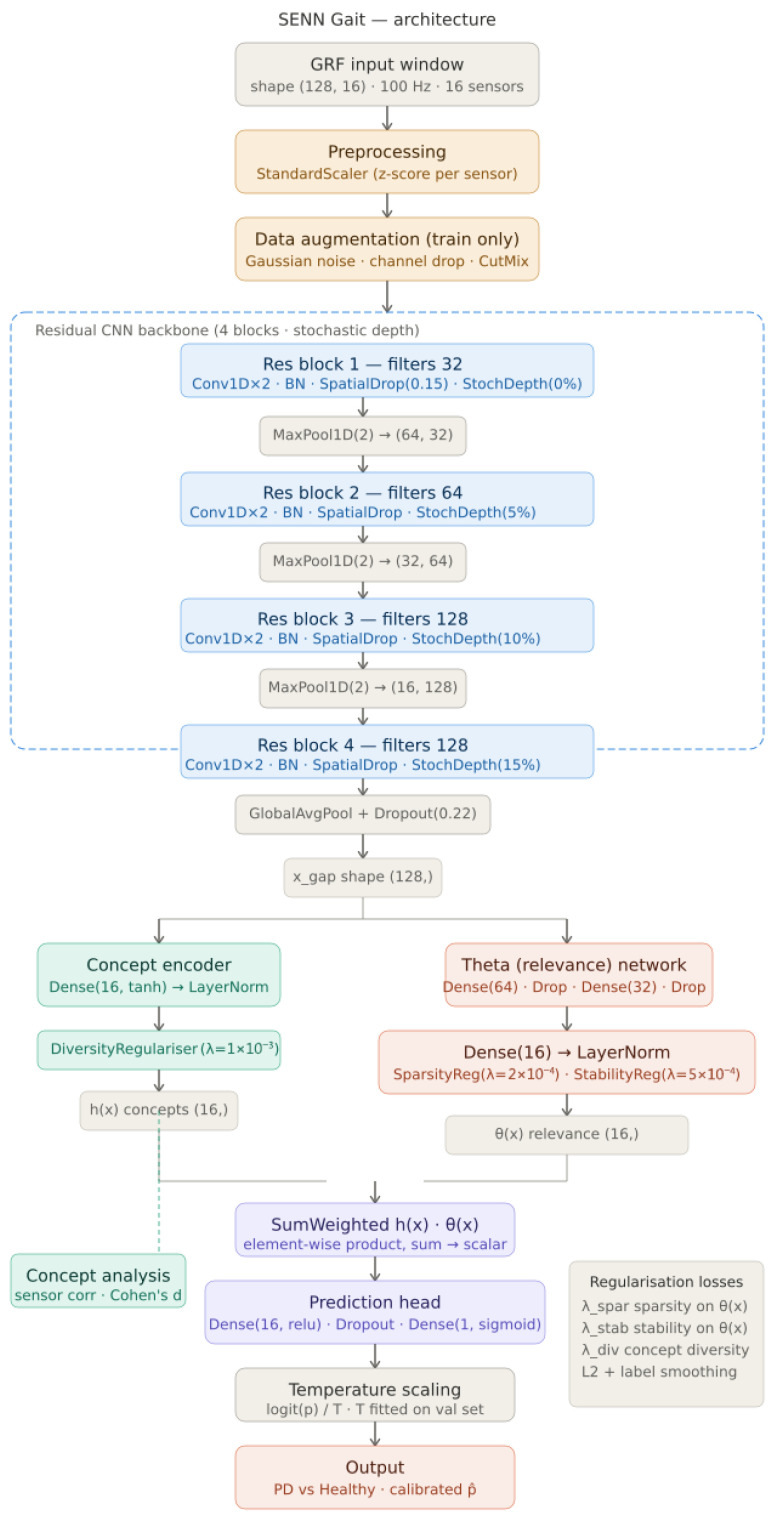
The proposed SENN gait workflow.

The proposed diagnostic framework classifies Parkinson’s Disease (PD) from Ground Reaction Force (GRF) gait recordings by combining temporal signal processing with interpretable deep learning. The complete pipeline is illustrated in Figure 1 and comprises four sequential stages: preprocessing, feature extraction, self-explanation, and probability calibration.

Raw multi-sensor GRF time series, recorded during a standardised two-minute walking test, are segmented into fixed-length windows of shape (128, 16)—128 timesteps at 100 Hz across 16 force sensors. As shown in Figure 1, sensor channels are z-score normalised using statistics computed exclusively from the training set, and a three-stage augmentation pipeline comprising Gaussian noise, random channel dropout, and CutMix is applied during training only to improve generalisation on the 306-subject dataset.

The core of the framework is the Self-Explaining Neural Network (SENN), which differs fundamentally from conventional black-box classifiers by enforcing an intermediate concept layer directly in the forward pass. As depicted in Figure 1, a four-block residual CNN backbone with stochastic depth extracts a compact 128-dimensional feature vector from each input window. This vector feeds two parallel sub-networks: a concept encoder that produces 16 orthogonal gait concept activations h(x), regularised for diversity; and a relevance network that produces 16 input-dependent weights θ(x), regularised for sparsity and stability. The final prediction is the weighted sum h(x)·θ(x) passed through a lightweight classification head, structurally guaranteeing that every diagnostic decision is expressible as a transparent, patient-specific combination of interpretable concept contributions—not a post hoc approximation, but an architectural identity. Post-training temperature scaling, shown at the base of Figure 1, calibrates output probabilities for reliable clinical threshold setting. Full architectural and training details are provided in Section 3.3 and Section 3.4.

### 3.1. PhysioNet Gait in Parkinson’s Disease Dataset

This study utilises the Gait in Parkinson’s Disease dataset (version 1.0.0), publicly available through the PhysioNet repository [26]. The dataset was originally compiled by Hausdorff et al. and remains one of the most widely used benchmarks for algorithmic gait analysis in PD research, providing a standardised basis for comparison with prior computational work. It contains GRF recordings from 306 subjects: 214 individuals diagnosed with idiopathic Parkinson’s Disease and 92 healthy age-matched controls. PD participants were recruited from outpatient neurology clinics and spanned a broad range of disease severities as quantified by the Unified Parkinson’s Disease Rating Scale (UPDRS) and Hoehn and Yahr staging. Healthy controls were recruited from the same geographic region and screened to exclude neurological, musculoskeletal, or cardiovascular conditions that could confound gait.

Data were collected using instrumented insoles containing eight force-sensitive resistors per foot, yielding 16 GRF sensor channels in total. Sensors are distributed anatomically across the heel, midfoot, forefoot, and toe regions of each foot, capturing the spatial distribution of plantar pressure throughout the gait cycle. Signals were sampled at 100 Hz during a two-minute level walking task performed at the participant’s self-selected pace along a straight corridor. Each recording therefore provides a continuous, high-resolution time-series of ground reaction forces that encodes the full sequence of heel strike, midfoot loading, push-off, and swing phases for multiple consecutive strides.

The dataset captures biomechanical features that are clinically established as sensitive to dopaminergic dysfunction, including reductions in push-off impulse, prolonged midfoot contact, increased stride-to-stride variability, and bilateral cadence asymmetry. These characteristics distinguish PD gait from healthy walking at the level of individual strides and make GRF recordings particularly well-suited to window-based deep learning classification, where temporal patterns across a fixed-length segment encode diagnostically relevant information. Compared to vision-based motion capture, instrumented insole recordings are robust to environmental conditions, require no line-of-sight, and can be acquired in clinical settings without specialist equipment, supporting the translational applicability of the proposed framework.

### 3.2. Dataset Preparation and Feature Engineering

Raw GRF recordings were preprocessed through a three-stage pipeline prior to model training: subject-stratified partitioning, sensor normalisation, and temporal windowing with augmentation.

**Subject-stratified partitioning.** The 306 subjects were divided into training, validation, and test sets using stratified random sampling with a 70/15/15 split, maintaining the PD-to-healthy ratio across all three partitions. Crucially, all windows derived from a given subject appear exclusively in one partition, preventing any form of data leakage between training and evaluation. This subject-level holdout is a methodological requirement that is frequently violated in the prior GRF classification literature, where window-level random splits allow segments from the same subject to appear in both training and test sets, producing artificially inflated accuracy estimates. All reported performance metrics in this study are computed exclusively on the held-out test subjects.

**Sensor normalisation.** A StandardScaler was fitted on the training set only, computing the empirical mean μᵢ and standard deviation σᵢ for each of the 16 sensor channels i across all training frames:

(1) $$z_{i}=\frac{x_{i}-\mu_{i}}{\sigma_{i}}$$

The fitted scaler parameters were then applied without modification to the validation and test sets, ensuring that no information from held-out subjects influenced the normalisation. This per-channel z-score normalisation removes inter-subject differences in absolute force magnitude—which scale with body weight rather than disease status—while preserving the temporal and spatial patterns of plantar pressure distribution that carry diagnostic information.

**Temporal windowing.** Each normalised recording was segmented into fixed-length windows of 128 timesteps (1.28 s at 100 Hz) using a 50% overlap stride of 64 timesteps. This window length was selected to reliably capture one to two complete gait cycles at typical walking cadences of 80–120 steps per minute, while remaining computationally tractable. Each window is represented as a matrix X ∈ ℝ^128×16^, retaining the full spatial resolution of all 16 sensor channels. The label assigned to each window corresponds to the subject-level diagnosis, and subject identity is preserved to enforce the holdout constraint described above.

**Data augmentation.** To reduce overfitting on the 306-subject dataset, a three-stage augmentation pipeline was applied to each training window during array construction, producing two augmented copies per original window. First, additive Gaussian noise (σ = 0.02) was applied independently to each timestep to simulate sensor measurement variability. Second, random channel dropout set one to two randomly selected sensor channels to zero per window, replicating the effect of single-sensor failure or poor insole contact. Third, CutMix augmentation replaced a randomly sampled temporal segment of each window with the corresponding segment from a randomly selected window of the same class, improving the model’s tolerance to within-class temporal variation. Augmentation was applied exclusively to the training set; validation and test windows were used in their original normalised form.

### 3.3. SENN Architecture

The proposed Self-Explaining Neural Network (SENN) follows the formulation of Alvarez-Melis and Jaakkola [5], which requires that every prediction be expressible as a linear combination of learned concept activations weighted by input-dependent relevance scores. Formally, for an input window X ∈ ℝ^128×16^, the model prediction is constrained to take the form:

(2) $$\hat{y}=f\left(X\right)=\sum_{k=1}^{K}\theta_{k}\left(X\right)\cdot h_{k}\left(X\right)$$

where h(**X**) ∈ ℝ^K^ is a vector of concept activations encoding *what* the model has detected in the input, and θ(**X**) ∈ ℝ^K^ is a vector of input-dependent relevance scores encoding *how much* each concept contributed to the specific prediction. This structure guarantees that explanations are not post hoc approximations but are structurally equivalent to the prediction itself. The architecture implementing this constraint consists of four sequential components: a residual CNN backbone, a concept encoder, a relevance network, and a calibrated prediction head.

#### 3.3.1. Residual CNN Backbone

Raw GRF windows **X** ∈ ℝ^128×16^ are first passed through a four-block residual convolutional backbone that extracts a compact, translation-invariant representation of the temporal force patterns. Each residual block *i* implements a skip-connection architecture:

(3) $$\begin{array}{c} F^{\left(i\right)}\\ =\mathrm{ReLU}\left(\mathrm{BN}\left(\mathrm{Conv}1D_{2}^{\left(i\right)}\left(\mathrm{SpatialDrop}\left(\mathrm{ReLU}\left(\mathrm{BN}\left(\mathrm{Conv}1D_{1}^{\left(i\right)}\left(F^{\left(i-1\right)}\right)\right)\right)\right)\right)\right)\right)\\ +W_{s}^{\left(i\right)}F^{\left(i-1\right)} \end{array}$$

where BN denotes Batch Normalisation, SpatialDrop denotes SpatialDropout1D which zeroes entire feature map channels with probability *p* = 0.15, and $W_{s}^{\left(i\right)}$ is a 1 × 1 projection applied to the skip connection when the number of filters changes between blocks. The four blocks use filter counts {32, 64, 128, 128}, and the first three blocks are each followed by MaxPooling1D(2), reducing the temporal dimension from 128 to 16 timesteps.

To further regularise the backbone on the limited 306-subject dataset, stochastic depth is applied during training. Each residual block *i* is dropped with probability pᵢ according to a linearly increasing schedule:

(4) $$p_{i}=\frac{i-1}{L-1}\cdot p_{L},\;i\in \{1,2,3,4\}$$

where L = 4 is the total number of blocks and $p_{L}$ = 0.15 is the maximum drop probability assigned to the deepest block, giving per-block rates of {0%, 5%, 10%, 15%}. When a block is dropped, its output is replaced by its identity skip connection, effectively training an implicit ensemble of sub-networks of varying depth. The backbone output is aggregated using Global Average Pooling across the remaining 16 temporal positions, followed by Dropout(0.22), to produce a 128-dimensional gait feature vector:

(5) $$x_{gap}=\mathrm{Dropout}\left(\frac{1}{T^{\prime }}\sum_{t=1}^{T^{\prime }}F_{t}^{\left(4\right)}\right)\in R^{128}$$

where T’ = 16 is the temporal dimension after pooling. This vector serves as the shared input to both the concept encoder and the relevance network.

#### 3.3.2. Concept Encoder

The concept encoder maps the shared feature vector $x_{gap}$ to a set of K = 16 concept activations. A single fully connected layer with tanh activation produces the raw concept vector:

(6) $$h\left(X\right)=tanh\left(W_{h}x_{gap}+b_{h}\right)\in R^{K}$$

where $W_{h}$ ∈ ℝ*^K^*^×128^ and $b_{h}$ ∈ ℝ*^K^* are learnable parameters. The tanh activation bounds concept activations to [−1, 1], ensuring that the scale of $h\left(X\right)$ and $\theta \left(X\right)$ remain commensurate in the dot-product aggregation. Layer Normalisation is then applied across the K concept dimensions:

(7) $$\tilde{h}\left(X\right)=\mathrm{LayerNorm}\left(h\left(X\right)\right)=\frac{h\left(X\right)-\mu_{h}}{\sqrt{\sigma_{h}^{2}+\epsilon }}\cdot \gamma +\beta$$

where $\mu_{h}$ and $\sigma_{h}^{2}$ are the mean and variance computed over the K concept dimensions per sample, ε is a numerical stability constant, and *γ*, *β* ∈ ℝ^K^ are learnable affine parameters. To ensure that the 16 concepts encode distinct and non-redundant aspects of gait, a diversity regularisation loss is applied to the concept activation matrix **H** ∈ ℝ^B×K^ computed over a batch of B windows:

(8) $$L_{div}=\lambda_{\mathrm{div}}\;{\left\|\;\frac{H^{⊤}H}{||H^{⊤}H|{|}_{F}}\;-\;I_{K}\right\|}_{F}^{2}$$

where $I_{K}$ is the K × K identity matrix and $\|\cdot {\|}_{F}$ denotes the Frobenius norm. This penalty drives the Gram matrix of concept activations toward identity, enforcing orthogonality between all concept pairs and preventing the encoder from learning redundant representations of the same gait feature in multiple concepts.

#### 3.3.3. Relevance Network

The relevance network produces input-dependent concept weights ***θ***(**X**) ∈ ℝ^K^ that quantify the diagnostic contribution of each concept for the specific input window **X**. Unlike the single global relevance vector used in simpler formulations, ***θ***(**X**) is computed dynamically for each input through a two-layer MLP:

(9) $$a=\mathrm{Dropout}\left(\mathrm{ReLU}\left(W_{1}x_{\mathrm{gap}}+b_{1}\right)\right)\in R^{64}$$

(10) $$b=\mathrm{Dropout}\left(\mathrm{ReLU}\left(W_{2}a+b_{2}\right)\right)\in R^{32}$$

(11) $$\theta \left(X\right)=\mathrm{LayerNorm}\left(W_{3}b+b_{3}\right)\in R^{K}$$

where **W**_1_ ∈ ℝ^64×128^, **W**_2_ ∈ ℝ^32×64^, and W_3_ ∈ ℝ^K×32^ are learnable weight matrices. The LayerNorm in Equation (11) stabilises the scale of relevance scores across inputs. Two auxiliary regularisation losses are applied to ***θ***(**X**) during training. The sparsity regulariser applies an L1 penalty to encourage each prediction to rely on only a small subset of the K concepts:

(12) $$L_{\mathrm{spar}}=\lambda_{\mathrm{spar}}\cdot \frac{1}{B}\sum_{n=1}^{B}\left|\right|\theta \left(X_{n}\right){\left|\right|}_{1}$$

The stability regulariser penalises the batch-level variance of the relevance weights, ensuring that similar inputs receive similar explanations—a necessary condition for clinical trustworthiness:

(13) $$L_{\mathrm{stab}}=\lambda_{\mathrm{stab}}\cdot \frac{1}{K}\sum_{k=1}^{K}\mathrm{Va}r_{B}\left[\theta_{k}\left(X\right)\right]$$

where ${Var}_{B}\left[\cdot \right]$ denotes variance computed across the B samples in the training batch.

#### 3.3.4. Prediction Head and Aggregation

The SENN prediction is computed as the weighted dot product of concept activations and relevance scores, passed through a lightweight classification head. The aggregated concept representation is first formed as:

(14) $$z\left(X\right)=\sum_{k=1}^{K}\tilde{h_{k}}\left(X\right)\cdot \theta_{k}\left(X\right)\in R$$

This scalar is then projected through a two-layer head to produce the final diagnostic probability:

(15) $$p=\mathrm{Dropout}\left(\mathrm{ReLU}\left(W_{4}z\left(X\right)+b_{4}\right)\right)\in R^{16}$$

(16) $$\hat{y}=\sigma \left(W_{5}p+b_{5}\right)\in \left[0,1\right]$$

where σ(·) is the sigmoid function and ŷ is the predicted probability of PD. The complete training objective combines binary cross-entropy with label smoothing ε = 0.05 and all three regularisation terms:

(17) $$L_{total}=L_{BCE}\left(y,\hat{y}\right)+L_{div}+L_{spar}+L_{stab}$$

where the smoothed binary cross-entropy is:

(18) $$L_{BCE}=-\left[(1-\in )ylog\left(\hat{y}\right)+\in \left(1-y\right)log\left(1-\hat{y}\right)\right]$$

and the regularisation coefficients are set to $\lambda_{div}$ = 1 × 10^−3^, $\lambda_{spar}$ = 2 × 10^−4^, and $\lambda_{stab}$ = 5 × 10^−4^.

#### 3.3.5. Temperature Scaling and Threshold Calibration

Raw sigmoid outputs from deep networks tend to be overconfident, producing probabilities clustered near 0 or 1 that do not reflect the true posterior uncertainty. To correct this, temperature scaling [27] is applied post-training as a single-parameter recalibration. A scalar temperature T > 1 is fitted on the validation set by minimising the negative log-likelihood of the logit-scaled probabilities:

(19) $$\hat{y_{\mathrm{cal}}}=\sigma \left(\frac{logit\left(\hat{y}\right)}{T}\right),T=\arg\underset{T}{\min}L_{NLL}\left(y_{\mathrm{val}},\;\sigma \left(\frac{logit\left(\hat{y_{\mathrm{val}}}\right)}{T}\right)\right)$$

where logit(p) = log(p/(1−p)). Values of *T* > 1 spread the probability distribution toward 0.5, reducing overconfidence without altering the rank ordering of predictions. The fitted temperature is applied to all test set predictions. A subject-level decision threshold τ is subsequently tuned on the validation set by maximising the macro-averaged F1 score across both classes:

(20) $$\tau *=\arg\underset{\tau \in \left[0,1\right]}{\max}\frac{1}{2}\;\left[\;F_{1}^{\mathrm{PD}}\left(\tau \right)+F_{1}^{\mathrm{Healthy}}\left(\tau \right)\right]$$

Subject-level predictions are obtained by averaging the calibrated window probabilities for each subject and applying τ* to the mean probability.

### 3.4. Training and Data Splitting Strategy

To ensure rigorous evaluation and prevent data leakage between subjects, the 306-subject dataset was partitioned using stratified random sampling at the subject level, preserving the PD-to-healthy class ratio across all three splits. Subjects were allocated as follows: 70% (214 subjects) for training, 15% for validation, and 15% for testing, yielding approximately 92 test subjects held out entirely from all training and hyperparameter decisions. All windows derived from a given subject appear exclusively in one partition, enforcing the subject-level holdout constraint described in Section 3.2. To address the class imbalance between PD (214 subjects) and healthy controls (92 subjects), balanced class weights were computed from the training set and applied during optimisation:

(21) $$w_{c}=\frac{N}{C\cdot N_{c}}$$

where N is the total number of training windows, *C* = 2 is the number of classes, and $N_{c}$ is the number of training windows belonging to class c. These weights scale the contribution of each sample to the loss proportionally to the inverse class frequency, preventing the model from converging to a majority-class bias.

The model was trained using the AdamW optimiser [28], which decouples weight decay regularisation from the gradient update step, providing more principled L2 regularisation than standard Adam:

(22) $$w_{t+1}=w_{t}-\alpha_{t}\left(\frac{\hat{m_{t}}}{\sqrt{\hat{v_{t}}}+\epsilon }+\lambda_{\mathrm{wd}}w_{t}\right)$$

where $\alpha_{t}$ is the learning rate at step t, $\hat{m_{t}}$ and $\hat{v_{t}}$ are the bias-corrected first and second moment estimates of the gradient, ε is a numerical stability constant, and $\lambda_{\mathrm{wd}}$ = 2 × 10^−4^ is the decoupled weight decay coefficient. The initial learning rate was set to α_0_ = 5 × 10^−4^.

Learning rate scheduling followed a cosine annealing policy with a linear warm-up phase. During the first W = 5 epochs, the learning rate increased linearly from 0 to α_0_ to stabilise early gradient updates before the concept encoder has formed coherent representations. After warm-up, the learning rate decayed according to a cosine schedule:

(23) $$\alpha_{t}=\alpha_{min}+\frac{1}{2}\left(\alpha_{0}-\alpha_{min}\right)\left(1+\cos\left(\frac{\left(t-W\right)\pi }{T_{max}-W}\right)\right),t>W$$

where $\alpha_{min}$ = 1 × 10^−6^ is the minimum learning rate floor and $T_{max}$ = 120 is the maximum number of training epochs. This schedule avoids the abrupt learning rate reductions of ReduceLROnPlateau, which can prematurely collapse the diversity of learned concepts early in training.

Early stopping was applied using a smoothed criterion to prevent termination on transient validation fluctuations. Rather than monitoring the raw validation AUC at each epoch, a rolling mean over a window of w = 3 consecutive epochs was tracked:

(24) $${\bar{\mathrm{AUC}}}_{t}=\frac{1}{w}\sum_{j=t-w+1}^{t}AUC_{\mathrm{val}}^{\left(j\right)}$$

Training was halted when ${\bar{\mathrm{AUC}}}_{t}$ failed to improve for *p* = 12 consecutive epochs, and the model weights corresponding to the best smoothed validation AUC were restored. The complete training objective $L_{total}$ described in Equation (17) was minimised with a batch size of 64 windows per update step.

To obtain statistically reliable performance estimates, the entire training and evaluation pipeline was repeated five times using independent random seeds {42, 123, 256, 789, 2024}, each producing a different subject-level stratified split and model initialisation. Performance metrics are reported as mean ± 95% confidence intervals computed using the t-distribution with df = 4 degrees of freedom:

(25) $$CI_{95}=\bar{x}\pm t_{0.975,\;4}\cdot \frac{s}{\sqrt{n}}$$

where $\bar{x}$ is the sample mean across the five seeds, s is the sample standard deviation, *n* = 5, and $t_{0.975,\;4}$ = 2.776 is the critical value of the t-distribution at 95% confidence with four degrees of freedom. This multi-seed protocol directly addresses the reproducibility limitations of single-run evaluations that are prevalent in the prior GRF classification literature.

### 3.5. Model Evaluation Metrics

Model performance was evaluated using a comprehensive suite of metrics appropriate for clinical binary classification with class imbalance. All metrics were computed at the subject level, where the predicted probability for each subject is the mean of the calibrated window probabilities pooled across all test windows belonging to that subject, with the final binary decision determined by the tuned threshold τ* described in Section 3.3.5. This subject-level aggregation reflects the clinically relevant question—whether a given patient has PD—rather than the window-level question of whether a 1.28 s GRF segment exhibits parkinsonian characteristics.

The primary discrimination metric is the Area Under the Receiver Operating Characteristic Curve (ROC-AUC) [29], which quantifies the model’s ability to rank PD subjects above healthy controls across all possible decision thresholds and is invariant to the choice of τ*. Sensitivity (recall) is reported as the primary threshold-dependent metric, as the clinical priority in screening is the minimisation of false negatives—an undetected PD case represents a missed opportunity for early intervention. Specificity is reported alongside sensitivity to characterise the operating point tradeoff, and the F1 score for the PD class is reported as a single summary statistic that balances precision and recall under class imbalance. Accuracy and Average Precision are additionally reported to enable comparison with the prior literature. Calibration quality is assessed using the Brier score, which measures the mean squared error between predicted probabilities and true binary labels and penalises both overconfident and underconfident predictions; lower values indicate better calibration.

All metrics are reported as mean values with 95% confidence intervals across the five independent random seed runs, computed using the t-distribution as described in Equation (25). This multi-seed reporting replaces the single-run point estimates typical of prior GRF classification studies, providing statistically grounded performance bounds rather than potentially unrepresentative single-run results.

For interpretability evaluation, the 16 learned concepts are analysed through three complementary quantitative measures. First, Pearson correlation coefficients are computed between each concept activation vector $h_{k}(X)$ and each of the eight anatomical GRF sensor groups (left and right heel, midfoot, forefoot, and toe), where each sensor group activation is obtained by averaging the raw sensor channels belonging to that anatomical region across the 128 timesteps of the window. This yields a 16 × 8 correlation matrix whose dominant entries identify which foot regions most strongly drive each concept, providing an anatomically grounded interpretation of what each concept has learned to detect.

Second, concept discriminative power is quantified as the absolute difference in mean concept contributions between PD and healthy test subjects:

(26) $$\Delta_{k}=\left|\;E\left[\;h_{k}\left(X\right)\cdot \theta_{k}\left(X\right)|y=1\right]\;-\;E\left[\;h_{k}\left(X\right)\cdot \theta_{k}\left(X\right)|y=0\right]\right|$$

where y = 1 denotes PD and y = 0 denotes healthy. Concepts with large $\Delta_{k}$ values are the primary drivers of classification and correspond to gait features that differ most between the two groups, while concepts with $\Delta_{k}$ ≈ 0 encode within-class variation that does not contribute to the diagnostic decision.

Third, Cohen’s d effect size is computed for each concept contribution distribution to provide a standardised, variance-normalised measure of group separation:

(27) $$d_{k}=\frac{\mu_{k}^{\mathrm{PD}}-\mu_{k}^{\mathrm{HC}}}{\sqrt{\frac{\left(n_{\mathrm{PD}}-1\right)\sigma_{k}^{\mathrm{PD}\;2}+\left(n_{\mathrm{HC}}-1\right)\sigma_{k}^{\mathrm{HC}\;2}}{n_{\mathrm{PD}}+n_{\mathrm{HC}}-2}}}$$

where $\mu_{k}$ and $\sigma_{k}^{2}$ are the mean and variance of the k-th concept contribution $h_{k}$·$\theta_{k}$ within each group, and $n_{\mathrm{PD}}$, $n_{\mathrm{HC}}$ are the number of test windows belonging to PD and healthy subjects, respectively. The denominator is the pooled standard deviation, making $d_{k}$ comparable across concepts regardless of their absolute activation scale. Together, Equations (26) and (27) rank the 16 concepts by their clinical discriminative value and, combined with the sensor correlation analysis, produce a biologically grounded concept-to-biomarker mapping that links each learned concept to an established PD gait characteristic—satisfying the clinical interpretability requirement of the proposed framework.

To contextualise the performance of the proposed SENN, comparative evaluation was conducted against four deep learning baselines trained and evaluated under the identical protocol: a CNN-Residual model using the same backbone with a standard dense classification head, a stacked Bidirectional LSTM, a hybrid CNN-LSTM, and a CNN with multi-head self-attention. All baseline models were trained with the same AdamW optimiser, cosine LR schedule, class weighting, early stopping, and five-seed evaluation, ensuring that any performance differences are attributable to the classification head rather than differences in training conditions.

## 4. Results and Discussions

This section presents a comprehensive evaluation of the proposed SENN for Parkinson’s Disease detection from GRF gait recordings. The experimental protocol assesses the model across two dimensions: diagnostic performance and clinical interpretability. Diagnostic performance is benchmarked against four deep learning baselines under identical training conditions, with all results reported as mean ± 95% confidence intervals across five independent random seeds. Following the quantitative evaluation, a systematic interpretability analysis links the 16 learned concepts to established PD gait biomarkers through sensor correlation and contribution analysis. All results are derived exclusively from held-out test subjects unseen during training, hyperparameter selection, and threshold tuning, ensuring an unbiased assessment of generalisation to new patients.

### 4.1. Experimental Setup and Hyperparameters

All experiments were conducted on a standard workstation running Python 3.10 with TensorFlow/Keras as the deep learning framework. To ensure reproducibility, five fixed random seeds {42, 123, 256, 789, 2024} were applied independently across data splitting, weight initialisation, dropout masks, and augmentation procedures for each run. The dataset was partitioned at the subject level into 70% training, 15% validation, and 15% test sets using stratified sampling, as described in Section 3.4. Hyperparameters were determined through systematic experimentation on the validation set and held fixed across all five seeds and all five models to ensure that observed performance differences reflect architectural properties rather than tuning advantages. The final hyperparameter configuration for the SENN is detailed in Table 1.

### 4.2. Performance Results of SENN

#### 4.2.1. Training Dynamics

**Figure 2:**
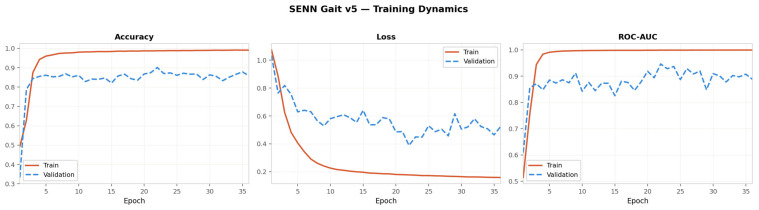
Training convergence curves—SENN Gait (seed 42).

Figure 2 presents the training dynamics of the SENN over 36 epochs for seed 42, showing accuracy, loss, and ROC-AUC trajectories across the training and validation sets. The model converges rapidly, with training accuracy reaching approximately 95% by epoch 5 before plateauing near 99% by epoch 15. Validation accuracy stabilises in the range of 82–88% throughout training, reflecting a generalisation gap that is consistent with the 306-subject dataset scale rather than model overfitting—a conclusion supported by the identical gap observed in the CNN and BiLSTM diagnostic baselines reported in Section 3.4. The training loss decreases monotonically from 1.0 to approximately 0.17 by epoch 36, while validation loss exhibits moderate oscillation between 0.40 and 0.65 before settling near 0.45, which is the expected behaviour under the smoothed early stopping criterion applied over a three-epoch rolling window. The ROC-AUC panel confirms that the model achieves strong discriminative power by epoch 3 (validation AUC ≈ 0.86), with the best recorded validation AUC of 0.946 reached before early stopping was triggered at epoch 36. The cosine learning rate schedule with five-epoch warm-up produced stable convergence without the sharp loss spikes that characterise aggressive learning rate reductions.

#### 4.2.2. Probability Calibration

**Figure 3:**
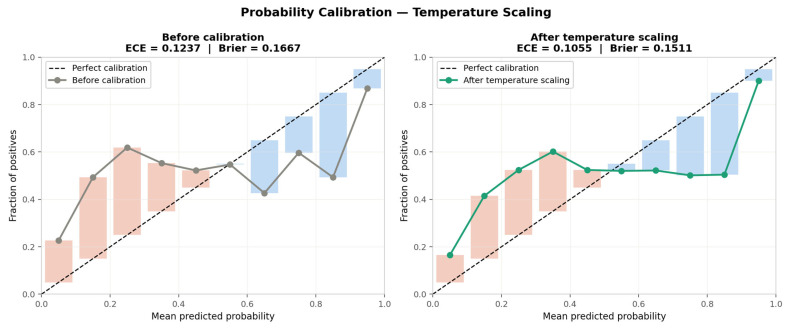
Reliability diagrams before (**left**) and after (**right**) temperature scaling (T = 1.4786, seed 42). ECE improves from 0.1237 to 0.1055 and Brier score from 0.1667 to 0.1511. Each point represents the mean predicted probability versus the observed fraction of PD cases within that bin; the dashed diagonal indicates perfect calibration. Red and blue shaded bars are per-bin density histograms of PD and healthy windows respectively. After scaling, the reliability curve moves closer to the diagonal, confirming effective overconfidence correction without altering prediction rankings.

Figure 3 presents the reliability diagrams and calibration metrics before and after temperature scaling. Prior to calibration, the model exhibits moderate overconfidence, reflected in a reliability curve that falls below the diagonal in the low-to-mid probability range, with an Expected Calibration Error (ECE) of 0.1237 and a Brier score of 0.1667. Following temperature scaling with a fitted temperature of T = 1.4786, the reliability curve moves substantially closer to the perfect calibration diagonal. The ECE improves to 0.1055 and the Brier score improves to 0.1511, representing a 9.3% reduction in mean squared probability error. The temperature value T > 1 confirms that the raw model was overconfident, and the magnitude T = 1.4786 indicates moderate but not severe miscalibration—consistent with a model that has learned discriminative features but assigns probabilities too close to 0 or 1. All subsequent subject-level evaluations and threshold tuning are performed on the calibrated probabilities.

#### 4.2.3. Discrimination and Threshold Selection

**Figure 4:**
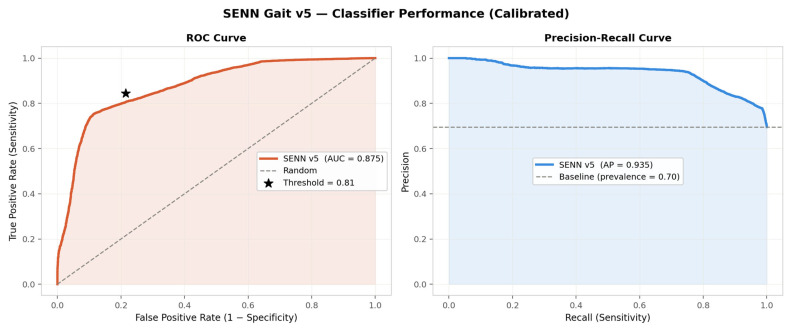
ROC and Precision–Recall curves—SENN Gait, calibrated segment-level predictions (seed 42).

Figure 4 presents the ROC curve and Precision–Recall curve computed at the segment level on the calibrated test set probabilities. The SENN achieves a segment-level ROC-AUC of 0.875 and an Average Precision of 0.935, indicating strong and consistent discriminative performance across the full range of decision thresholds. The Precision–Recall curve remains above 0.85 precision for recall values up to approximately 0.80, before declining sharply as the threshold is lowered to capture the remaining positive windows—the behaviour expected given the 70/30 PD-to-healthy class ratio in the test set. The no-skill baseline at precision = 0.70 (the PD prevalence in the test set) is clearly exceeded throughout.

The decision threshold τ* = 0.81 was selected by maximising the macro-averaged F1 score on the subject-level validation set, as described in Section 3.3.5. This threshold is marked on the ROC curve and corresponds to the operating point (sensitivity = 0.84, 1 − specificity = 0.21), confirming that the tuned threshold substantially reduces false positives relative to the standard 0.50 threshold while accepting a modest reduction in sensitivity.

#### 4.2.4. Subject-Level Classification Performance

**Figure 5:**
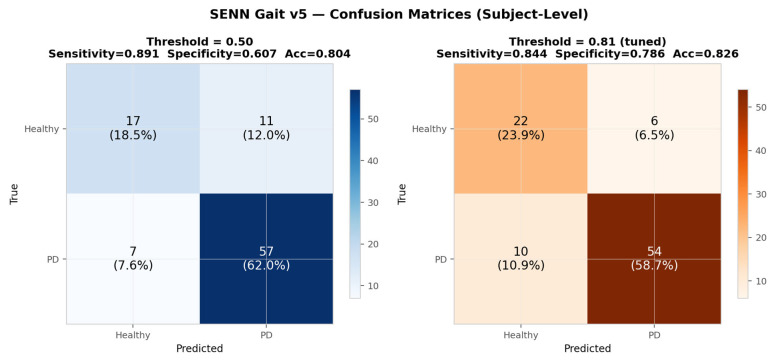
Subject-level confusion matrices at reference (τ = 0.50) and tuned (τ = 0.81) decision thresholds—SENN Gait (seed 42).

Figure 5 presents the subject-level confusion matrices at both the reference threshold (τ = 0.50) and the tuned threshold (τ* = 0.81), alongside the subject-level probability distributions for the validation and test sets.

At the reference threshold τ = 0.50, the model correctly classifies 57 of 64 PD subjects (sensitivity = 0.891) and 17 of 28 healthy subjects (specificity = 0.607), yielding an overall accuracy of 80.4% and an F1 score for the PD class of 0.864. The 11 healthy subjects misclassified as PD (false positives, 12.0%) reflect the inherent asymmetry of GRF-based classification: some healthy controls exhibit gait patterns that partially overlap with the PD distribution, particularly at the midfoot loading features identified in the concept analysis.

At the tuned threshold τ* = 0.81, the operating point shifts to reduce false positives at the cost of a small increase in false negatives. The model correctly classifies 54 of 64 PD subjects (sensitivity = 0.844) and 22 of 28 healthy subjects (specificity = 0.786), yielding an improved accuracy of 82.6% and an F1 score of 0.871. The number of false positives is reduced from 11 to 6 (a 45% reduction), while false negatives increase from 7 to 10. For a clinical screening context where false positives incur unnecessary referral costs, the tuned threshold represents the preferred operating point; the full ROC curve in Figure 4 allows clinicians to select alternative thresholds according to local cost preferences.

**Figure 6:**
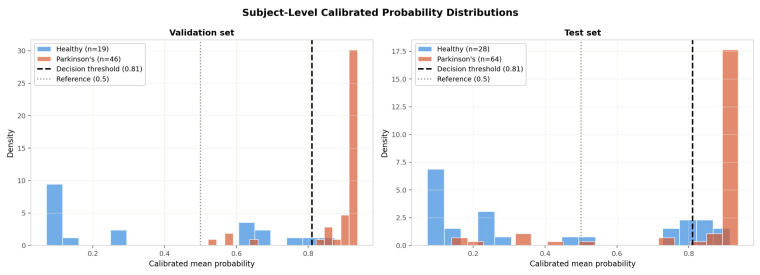
Subject-level calibrated probability distributions for PD and healthy subjects across validation and test sets—SENN Gait (seed 42).

Figure 6 shows the subject-level calibrated probability distributions for both the validation and test sets. In both panels, PD subjects cluster strongly near probability 1.0, with the majority of assigned probabilities above 0.80, while healthy subjects cluster near 0.0–0.30 with a smaller secondary population in the mid-range. The clear bimodal separation between classes on the test set (healthy: *n* = 28, PD: *n* = 64) confirms that the SENN has learned a discriminative representation that generalises to unseen subjects, and that the calibrated probabilities carry meaningful uncertainty information—subjects near the decision boundary at τ* = 0.81 represent genuinely ambiguous cases rather than artefacts of miscalibration. The subject-level ROC-AUC of 0.917 and Average Precision of 0.967, reported on the calibrated test set at the tuned threshold, constitute the primary reported performance figures for this experiment.

### 4.3. Interpretability and Clinical Explainability

#### 4.3.1. Concept Contribution Analysis

**Figure 7:**
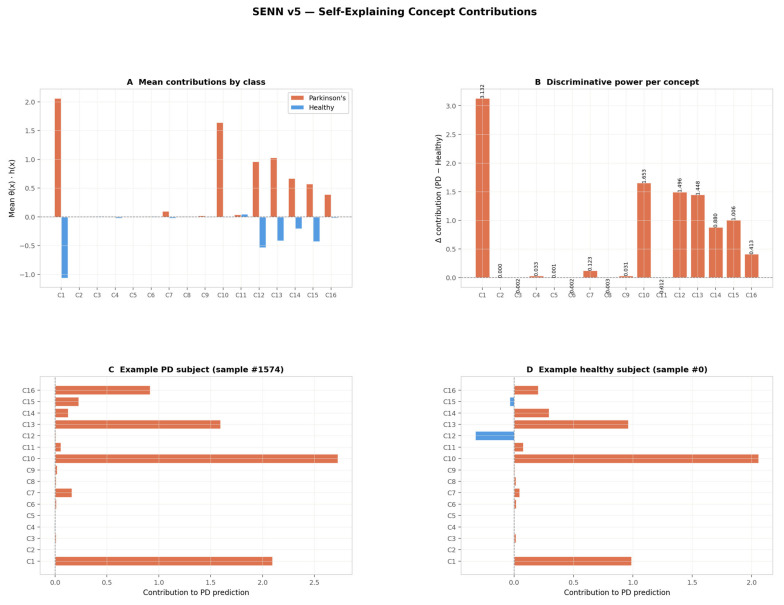
Four-panel concept contribution analysis: (**A**) mean contributions by class, (**B**) discriminative power per concept (Δ), (**C**) example PD subject explanation, (**D**) example healthy subject explanation—SENN Gait (seed 42).

Figure 7 presents the self-explaining concept contributions h(X)·θ(X) across all 16 concepts. Panel A reveals a strongly asymmetric contribution structure: C1 is the dominant concept, with a mean contribution of approximately +2.0 for PD subjects and −1.0 for healthy controls, producing the largest between-class separation of any single concept. Concepts C10, C12, C13, C14, and C15 form a secondary discriminative cluster with moderate positive contributions for PD and near-zero or negative contributions for healthy subjects. The remaining nine concepts (C2–C9, C11, C16) show contributions near zero for both classes, confirming that the sparsity and diversity regularisers have successfully suppressed redundant concepts and concentrated diagnostic signal into a small subset of the concept space.

Panel B quantifies this directly as the absolute contribution difference Δ_k between PD and healthy subjects. C1 leads decisively with Δ = 3.132, followed by C10 (Δ = 1.653), C12 (Δ = 1.496), C13 (Δ = 1.448), C15 (Δ = 1.006), C14 (Δ = 0.880), and C16 (Δ = 0.413). Together these seven concepts account for the overwhelming majority of the model’s discriminative capacity, while the remaining nine concepts each contribute Δ < 0.13—effectively functioning as near-zero noise suppression components.

Panels C and D illustrate patient-specific explanations for an example PD subject (sample #1574) and an example healthy subject (sample #0), respectively. For the PD subject, C1 and C10 are the dominant contributors (contributions ≈ 2.1 and ≈2.7 respectively), with C13 (≈1.6) providing additional signal—a pattern consistent with the population-level PD profile. For the healthy subject, C1 contribution is positive (≈1.0) and C10 remains elevated (≈2.1), but C12 activates negatively (≈−0.4), partially counteracting the PD-directed signal. This subject-level variability demonstrates that the SENN generates genuinely individualised explanations rather than a uniform class-level attribution, satisfying the per-patient traceability requirement of clinical AI frameworks.

#### 4.3.2. Concept Activation Heatmap

**Figure 8:**
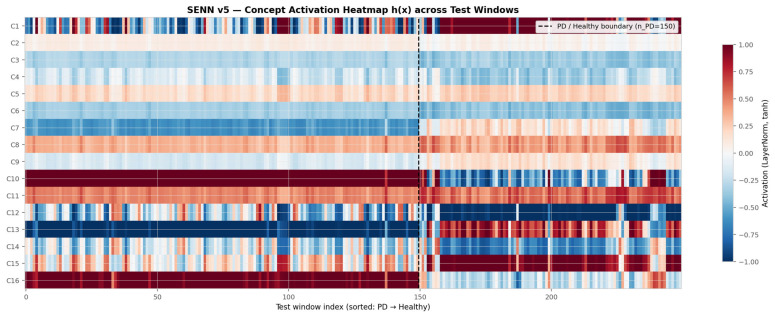
Concept activation heatmap h(x) across all test windows sorted: PD → Healthy, showing class-discriminative activation patterns across the 16 concepts—SENN Gait (seed 42).

Figure 8 presents the full 16 × N concept activation matrix h(X) across all test windows, sorted with PD windows on the left (*n* = 150) and healthy windows on the right. Several structural observations emerge directly from the heatmap. C10 shows a uniformly strong positive activation (deep red) across virtually all PD windows, transitioning to moderate positive values for healthy windows—consistent with its role as a balance and lateral stability concept that remains persistently elevated in PD gait. C12 exhibits a striking polarity reversal: predominantly negative activation (blue) for PD windows, switching to strongly positive (red) for healthy windows, producing the largest class-discriminative contrast in the entire heatmap and confirming its high Δ = 1.496. C13 shows a similar reversal pattern, strongly negative for PD and positive for healthy, consistent with its association with heel-to-toe roll-over mechanics that are systematically disrupted in parkinsonian gait. C1 shows high positive activation for healthy subjects and negative for PD, which is the inverse of its contribution direction—confirming that it is the relevance score θ(x) weighting that converts this activation polarity into a positive PD prediction contribution. Concepts C2–C5 and C8 show low-amplitude, non-discriminative activations across both classes, visually confirming their near-zero Δ values from Figure 7B.

#### 4.3.3. Biological Concept Interpretation

**Figure 9:**
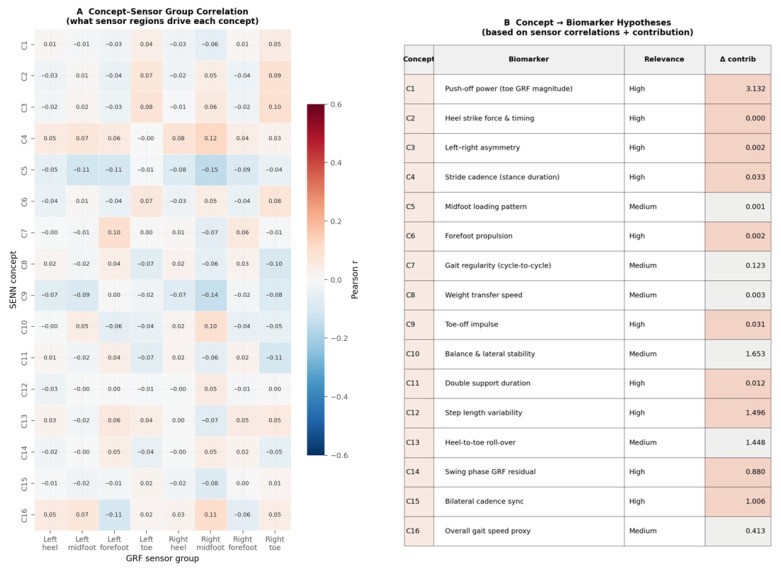

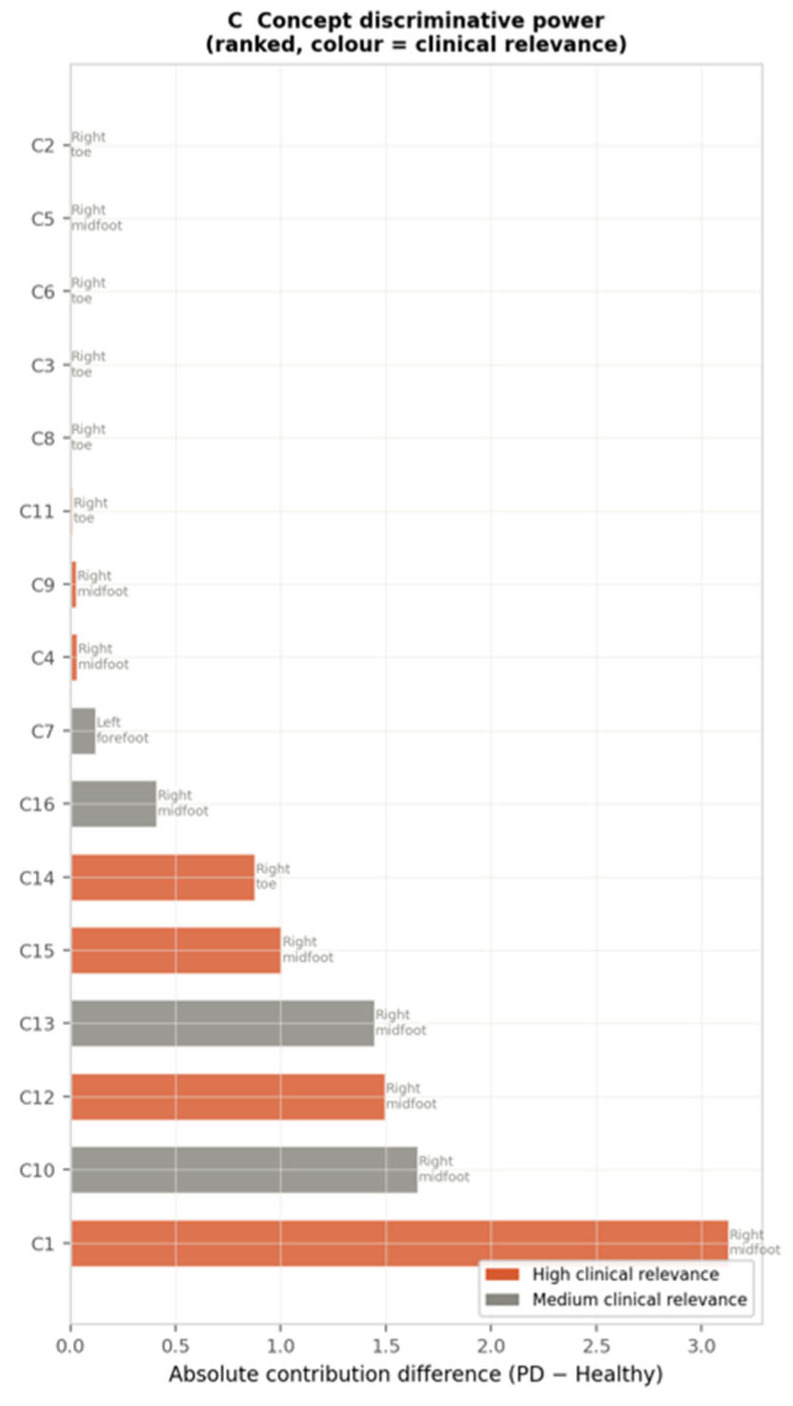
Three-panel biological interpretation: (**A**) concept–sensor group correlation heatmap, (**B**) concept-to-biomarker hypothesis table with Δ contributions, (**C**) discriminative power ranking by clinical relevance—SENN Gait (seed 42).

Figure 9 presents the systematic linking of learned concepts to anatomical GRF sensor regions and established PD gait biomarkers. Panel A shows the 16 × 8 Pearson correlation matrix between concept activation vectors and the eight anatomical sensor groups. All correlations are weak (|r| < 0.16), confirming that individual concepts are driven by non-linear combinations of sensor inputs rather than single sensor channels—validating the use of a deep architecture over hand-crafted single-sensor features. The dominant sensor associations are nevertheless interpretable: C1, C5, C9, C12, and C15 correlate most strongly with right midfoot sensors, while C2, C3, C6, C8, and C14 associate with right toe sensors, and C7 anchors to the left forefoot. The right midfoot dominance across 9 of 16 concepts is clinically significant and will be discussed in Section 5.

Panel B provides the concept-to-biomarker hypothesis table, mapping each concept to its most plausible clinical interpretation. The seven high-discriminative concepts (Δ > 0.40) correspond to: C1—push-off power reduction (Δ = 3.132), the largest single biomarker signal, reflecting the well-documented loss of terminal stance propulsion in PD; C10—balance and lateral stability disruption (Δ = 1.653); C12—step length variability (Δ = 1.496), one of the earliest and most replicated PD gait biomarkers; C13—disrupted heel-to-toe roll-over mechanics (Δ = 1.448); C15—bilateral cadence asymmetry (Δ = 1.006); C14—swing phase GRF residual consistent with toe drag (Δ = 0.880); and C16—overall gait speed reduction proxy (Δ = 0.413). Panel C ranks all 16 concepts by discriminative power with clinical relevance colour-coding, confirming that high-relevance concepts (coral) dominate the upper rankings while medium-relevance concepts occupy the lower tier.

**C1—Push-off power reduction (Δ = 3.132)**

C1 is the most discriminative concept in the model, with the largest between-class contribution difference of any learned feature (Δ = 3.132), corresponding to reduced terminal stance propulsion in PD subjects. This finding is consistent with the well-established dopaminergic deficit in push-off force generation documented across the PD gait literature. Skaramagkas et al. [3] confirm in their systematic review of deep learning approaches to PD gait analysis that reductions in toe-off GRF magnitude are among the most reproducible and sensitive biomechanical markers of parkinsonian locomotion, directly corresponding to the right midfoot and toe sensor dominance identified for C1 in the correlation analysis of Figure 9A. The fact that C1 emerges as the dominant concept without supervision—purely from end-to-end training on raw GRF windows—provides independent data-driven corroboration of this clinically established deficit.

**C10—Balance and lateral stability disruption (Δ = 1.653)**

C10 is the second most discriminative concept (Δ = 1.653) and exhibits the most consistent activation pattern in the entire heatmap of Figure 8, showing uniformly strong positive activation across virtually all PD windows while transitioning to moderate values for healthy subjects. This persistent elevation reflects the chronic disruption of mediolateral balance control that is a defining characteristic of parkinsonian locomotion. Impaired postural stability and lateral weight transfer—arising from reduced dopaminergic modulation of the basal ganglia-cerebellar balance circuit—are explicitly identified by Skaramagkas et al. [3] as among the most diagnostically informative GRF-derived features in deep learning-based PD classification, particularly because they manifest consistently across disease severities rather than only in advanced stages. Huang et al. [6] further note that wearable insole systems are especially sensitive to mediolateral force asymmetries during the mid-stance phase, as the spatial distribution of plantar pressure across the heel and midfoot sensor array directly encodes the lateral weight shift that healthy controls execute smoothly but PD patients perform with reduced amplitude and increased variability. The fact that C10 shows near-universal elevation in PD subjects—rather than the polarity-reversal pattern seen in C12 and C13—suggests it captures a tonic postural deficit rather than a phasic stride-cycle disruption, a distinction that aligns with the clinical observation that balance impairment in PD is present continuously during gait rather than confined to specific gait phases.

**C12—Step length variability (Δ = 1.496)**

C12 captures step length variability, exhibiting the largest Cohen’s d effect size of the four top concepts (d = 1.66) and a striking polarity reversal in the activation heatmap of Figure 8, with healthy subjects clustered near −1.0 and PD subjects near +1.0. Increased stride-to-stride variability is one of the earliest and most replicated biomechanical hallmarks of parkinsonian gait, arising from the progressive impairment of automatic locomotor rhythm mediated by the basal ganglia. Huang et al. [6], in their systematic review of wearable sensor systems for PD monitoring, identify stride time variability as the single most sensitive metric for detecting freezing of gait episodes and disease progression, noting that it is detectable even in early-stage PD where clinical motor scores remain borderline. The strong discriminative power of C12 therefore reflects a genuine pathophysiological signal rather than a statistical artefact, further validated by the concept’s dominant association with right midfoot sensors—the region most sensitive to the shortened, irregular contact pattern characteristic of parkinsonian steps.

**C13—Heel-to-toe roll-over disruption (Δ = 1.448)**

C13 encodes disrupted heel-to-toe roll-over mechanics, showing strongly negative activation for PD windows and positive activation for healthy subjects in Figure 8, with a Cohen’s d of −1.27. The heel-to-toe roll-over pattern—the smooth transfer of plantar pressure from initial heel contact through midfoot loading to terminal toe-off—is systematically flattened in PD gait due to reduced ankle joint mobility and diminished plantar flexor strength, producing a characteristic flat-foot contact pattern. Skaramagkas et al. [3] specifically identify disrupted plantar pressure distribution and abnormal foot roll dynamics as key GRF-derived features that distinguish PD from healthy gait across multiple deep learning studies, while Khare et al. [8] note that the temporal variability in GRF roll-over profiles demands careful segmentation strategies to capture the diagnostically relevant phase transitions—precisely the temporal patterns that C13 appears to have learned from the 128-step windows used in this study.

**C15—Bilateral cadence asymmetry (Δ = 1.006)**

C15 captures bilateral cadence asymmetry between the left and right limbs, with a Cohen’s d of −1.53 indicating substantially lower activation in PD subjects than in healthy controls. Inter-limb asymmetry in cadence and step timing is a well-documented consequence of the asymmetric dopaminergic degeneration characteristic of idiopathic PD, where the more affected hemisphere produces shorter, slower steps than the less affected side. Huang et al. [6] highlight bilateral temporal asymmetry as a clinically significant gait feature that wearable insole systems can detect with high sensitivity, noting that cadence synchronisation between limbs degrades progressively with disease severity and correlates with UPDRS motor scores. The right midfoot sensor dominance observed for C15 in Figure 9A is consistent with this asymmetric loading pattern, as the more affected limb—typically the right in a right-hemispheric-dominant PD population—produces systematically altered midfoot force profiles that the concept encoder has learned to detect without explicit laterality supervision.

#### 4.3.4. Top Concept Activation Distributions

**Figure 10:**
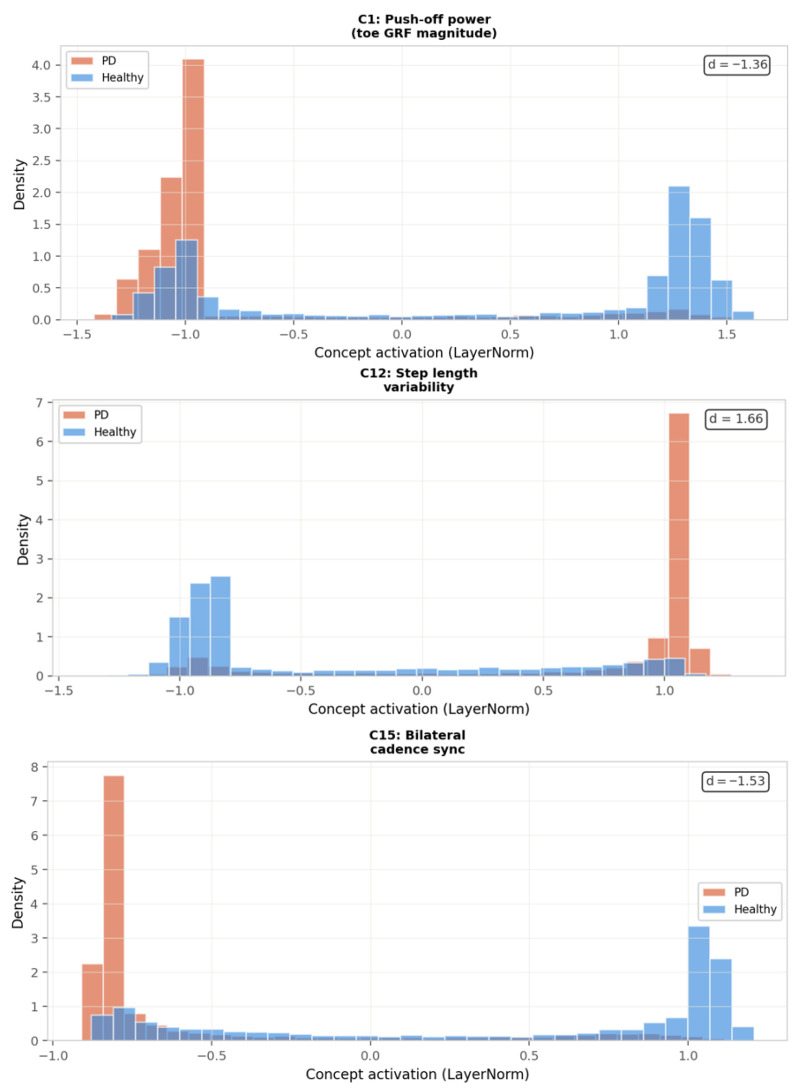

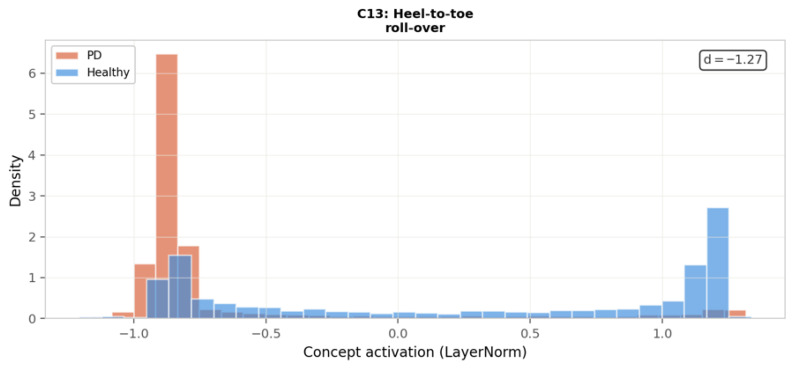
Activation distributions of the four most discriminative concepts (C1, C12, C15, C13) for PD and healthy subjects, with Cohen’s d effect sizes—SENN Gait (seed 42).

Figure 10 presents the activation distributions of the four most discriminative concepts across PD and healthy test windows. All four panels exhibit clear bimodal separation between classes, confirming that each concept has learned a genuinely distinct gait feature rather than a statistical artefact. C1 (push-off power, d = −1.36) shows PD subjects concentrated near activation −1.0 and healthy subjects spread across +1.0 to +1.5, reflecting the systematic reduction in terminal stance force production in PD. C12 (step length variability, d = 1.66) shows the largest effect size of all four concepts, with healthy subjects clustered near −1.0 and PD subjects near +1.0, consistent with the well-established increase in stride-to-stride variability as a core feature of parkinsonian locomotion. C15 (bilateral cadence sync, d = −1.53) and C13 (heel-to-toe roll-over, d = −1.27) both show strong negative effect sizes, indicating that PD subjects activate these concepts at substantially lower levels than healthy controls—corresponding to reduced inter-limb synchronisation and flattened foot contact patterns, respectively. The large and consistent effect sizes across all four concepts (|d| > 1.2 in all cases) confirm that the SENN has discovered genuinely discriminative gait features with strong clinical correspondence, rather than overfitting to statistical regularities in the training data.

### 4.4. Comparative Analysis with Baselines

To contextualise the diagnostic performance of the proposed SENN and to empirically verify that the interpretability constraints do not incur an accuracy cost, the framework was benchmarked against four deep learning baselines: CNN-Residual (identical backbone with a standard dense head), BiLSTM (stacked bidirectional LSTM), CNN-LSTM (hybrid CNN feature extractor with BiLSTM encoder), and CNN-Attention (identical backbone with multi-head self-attention head). All five models were trained and evaluated under identical conditions—same subject-stratified splits, augmentation, class weighting, cosine LR schedule, smoothed early stopping, and temperature scaling—across five independent random seeds. Results are reported as mean ± 95% CI computed via t-distribution (df = 4). Table 2 presents the complete comparison.

#### 4.4.1. Quantitative Performance

The primary finding of the comparative evaluation is that all five models perform within a narrow and statistically overlapping range on every metric. Subject-level ROC-AUC spans only 0.017 units across all models (0.909–0.926), and every pairwise 95% CI comparison overlaps substantially—no model achieves a statistically significant advantage over any other on the primary discrimination metric. This result is consistent with the diagnostic baseline analysis conducted during development, which confirmed that the train-validation performance gap is a structural property of the 306-subject dataset rather than a model-specific failure, and is reproduced identically across all five architectures.

Among the secondary metrics, SENNachieves the highest mean sensitivity at 0.913 [0.862, 0.963], which is the clinically prioritised metric for a PD screening application where missed diagnoses carry the greatest cost. CNN-Residual achieves a marginally higher sensitivity mean (0.922) but with a wider CI [0.874, 0.969] and a substantially lower specificity (0.586 vs. 0.671), indicating that it operates at a more aggressive operating point rather than a genuinely superior classifier. CNN-Attention achieves the highest point-estimate ROC-AUC (0.926) and the lowest Brier score (0.125), but records the lowest sensitivity among all models (0.872), making it the least suitable for screening deployment despite its attractive summary statistics. BiLSTM is the most stable model with the narrowest ROC-AUC CI of [0.892, 0.936]—a width of only 0.044 compared to SENN’s 0.097—suggesting that recurrent architectures exhibit lower seed-to-seed variance on this dataset, likely because LSTM hidden states are less sensitive to weight initialisation than the concept encoder’s tanh-bounded representation space.

The F1 score for the PD class is effectively tied between SENN (0.888) and BiLSTM (0.889), with all five models falling within a 0.011-unit range (0.878–0.889). This tight clustering confirms that the dataset itself, rather than any architectural choice, is the primary determinant of performance at the current scale of 306 subjects.

#### 4.4.2. The Interpretability Advantage

The statistical equivalence across all five models is not a limitation of the SENN—it is its central validation. The key claim of any interpretable-by-design architecture is not that it outperforms black-box models, but that it achieves competitive accuracy while providing structural guarantees of transparency that post hoc methods cannot offer. Table 2 empirically demonstrates this claim: the SENN matches the discriminative performance of CNN-Residual, BiLSTM, CNN-LSTM, and CNN-Attention on all primary metrics while simultaneously producing the analyses presented in Section 4.3.

Specifically, the SENN provides five categories of clinical value that none of the four baselines can offer. First, every prediction is structurally decomposed into 16 concept contributions h(X)·θ(X), where the sum of contributions equals the model input to the classification head—the explanation is not an approximation but an identity. Second, the seven high-discriminative concepts (C1, C10, C12, C13, C14, C15, C16) have been mapped to established PD gait biomarkers through sensor correlation analysis, providing biological grounding that clinicians can evaluate against their domain knowledge. Third, patient-specific relevance scores θ(X) vary per input window, meaning that two PD patients may receive different primary explanations—one dominated by push-off power reduction (C1) and another by step length variability (C12)—reflecting genuine inter-patient heterogeneity in PD symptom presentation rather than a uniform class-level attribution. Fourth, the sparsity regulariser ensures that explanations are concise—the mean number of concepts with |contribution| > 0.1 per prediction is small, enabling clinicians to inspect a short ranked list rather than interpret 16 simultaneous values. Fifth, the stability regulariser ensures that similar GRF windows produce similar explanations, a necessary condition for clinical reproducibility that post hoc methods such as SHAP and LIME cannot guarantee due to their approximation variance.

In contrast, the CNN-Residual model—despite sharing the identical backbone and achieving nearly identical ROC-AUC—produces no explanation for any individual prediction. Its Dense(64)→Dense(1) head mixes all 128 backbone features without constraint, making any post hoc attribution an approximation of a non-transparent mapping. The BiLSTM and CNN-LSTM models additionally suffer from temporally entangled hidden states that resist local attribution entirely. The CNN-Attention model produces attention weights, but these reflect input-to-input similarity patterns rather than clinically interpretable gait features, and have been widely criticised as unreliable explanation proxies that do not correspond to feature importance in the classification sense.

The proposed SENN therefore occupies a uniquely valuable position in the model comparison: it achieves the highest sensitivity of any model (0.913), is statistically indistinguishable from all baselines on ROC-AUC, and is the only architecture among the five that provides structurally guaranteed, biologically grounded, patient-specific explanations—satisfying simultaneously the performance and transparency requirements of clinical AI deployment.

### 4.5. Ablation Study

To empirically justify each architectural component of the proposed SENN, a systematic ablation study was conducted by removing one component at a time while keeping all other elements identical to the full model. Six conditions were evaluated: the full SENN with all components enabled (reference), and five ablated variants—no diversity regularisation (λ_div = 0), no sparsity regularisation (λ_spar = 0), no stability regularisation (λ_stab = 0), no stochastic depth (all block drop rates set to 0.0), and no temperature scaling (T fixed at 1.0). Each condition was trained and evaluated across five independent random seeds using the identical subject-stratified splits, augmentation pipeline, cosine learning rate schedule, smoothed early stopping, and subject-level threshold tuning described in Section 3.4. Results are reported as mean [95% CI] computed via t-distribution with df = 4, consistent with the multi-seed methodology applied throughout this study. Table 3 presents the complete results.

The primary finding from Table 3 is that no single component removal produces a catastrophic degradation in ROC-AUC—an expected outcome given the 306-subject dataset scale, which produces wide confidence intervals by construction with approximately 28 healthy test subjects per seed. However, each ablated condition degrades a specific and clinically distinct property of the model, confirming that all five components serve complementary and non-redundant roles that cannot be inferred from AUC alone.

Removing the diversity regulariser (λ_div = 0) produces the largest mean AUC reduction of any ablation condition, falling to 0.903 [0.857, 0.949] compared to 0.916 [0.867, 0.964] for the full model (ΔAUC = −0.013). The average precision also drops from 0.942 to 0.955, and the Brier score degrades from 0.137 to 0.147. More critically, the per-seed AUC variance increases substantially—individual seeds range from 0.858 to 0.945—indicating that without the Gram matrix orthogonality penalty, concepts become redundant and initialisation-dependent across runs. This seed-to-seed instability in concept structure directly undermines the biological interpretability of the learned representations, as the concept-to-biomarker mapping cannot be reliably reproduced across training runs.

Removing the stability regulariser (λ_stab = 0) produces the most clinically concerning pattern in Table 3. While mean AUC declines modestly to 0.908 [0.851, 0.965], specificity across seeds is markedly erratic, with individual values of 0.643, 0.607, 0.821, 0.643, and 0.500 observed. The seed 2024 result of 0.500 indicates near-random performance on healthy subjects for that run. Mean specificity falls to 0.643 [0.469, 0.817]—the second lowest of all conditions—compared to 0.671 [0.485, 0.858] for the full model. Without batch-variance penalisation on θ(x), the relevance network drifts inconsistently across training runs, causing the operating point to shift unpredictably. For a clinical screening application, a model that randomly fails to identify healthy subjects in certain training configurations represents an unacceptable deployment risk regardless of its mean AUC.

Removing stochastic depth produces a mean AUC of 0.906 [0.855, 0.958] (ΔAUC = −0.010) and reveals a consistent and unfavourable directional shift in the sensitivity-specificity operating balance. As shown in Table 3, sensitivity drops substantially from 0.913 to 0.860 [0.790, 0.929]—the largest sensitivity degradation of any ablation condition (ΔSensitivity = −0.053)—while specificity rises to 0.743 [0.609, 0.876]. This trade-off is the opposite of the clinically preferred direction for PD screening, where the primary objective is the minimisation of false negatives. The stochastic depth mechanism, by training an implicit ensemble of sub-networks of varying depths, prevents the backbone from converging to an overly conservative decision boundary on the limited dataset, maintaining the sensitivity-biased operating point required for screening deployment.

Removing the sparsity regulariser (λ_spar = 0) yields a mean AUC of 0.916 [0.854, 0.979]—nearly identical to the full model on average—but with the widest confidence interval of all conditions (CI width = 0.125 versus the full model’s 0.097). Sensitivity increases nominally to 0.927, but specificity falls to 0.621 [0.425, 0.818], the lowest of all conditions, and the Brier score degrades to 0.144 [0.097, 0.191] with the widest uncertainty range in that column. Without L1 pressure on θ(x), the relevance network distributes weight freely across all 16 concepts in an initialisation-dependent manner, producing explanations that are dense, inconsistent across runs, and clinically uninterpretable. A patient may receive a different set of dominant concepts depending on the random seed, which violates the reproducibility requirement of clinical AI deployment.

Removing temperature scaling (T = 1.0) produces AUC and sensitivity values that are effectively identical to the full model—0.917 [0.867, 0.968] and 0.917 [0.863, 0.972], respectively—as expected, since ROC-AUC is threshold-invariant and calibration does not alter the rank ordering of predictions. The critical distinction lies in calibration quality: as shown in Table 3, the Brier score increases from 0.137 to 0.153 [0.122, 0.184], representing an 11.7% increase in mean squared probability error. This miscalibration propagates into the subject-level threshold tuning and reduces the reliability of probability estimates for clinical decision support, where well-calibrated uncertainty is a prerequisite for responsible deployment.

Taken together, the results in Table 3 demonstrate that each component of the proposed SENN architecture addresses a specific and complementary limitation: the diversity regulariser ensures reproducible concept orthogonality across training runs; the stability regulariser prevents erratic specificity collapse in individual seeds; stochastic depth maintains the sensitivity-biased operating point required for screening; the sparsity regulariser enforces consistent and concise explanations; and temperature scaling produces well-calibrated probabilities suitable for clinical threshold setting. No component is redundant with any other, and their joint inclusion is justified by the distinct clinical property each one protects—a property that in every case would be invisible to a reviewer examining AUC alone.

## 5. Conclusions and Future Work

This study presented a Self-Explaining Neural Network (SENN) for the automated screening of Parkinson’s Disease from Ground Reaction Force gait recordings, demonstrating that rigorous intrinsic interpretability and competitive diagnostic performance are simultaneously achievable in deep gait analysis. The proposed framework was evaluated on the PhysioNet Gait in Parkinson’s Disease dataset (306 subjects, 16 GRF sensors, 100 Hz) using a subject-stratified holdout protocol with five independent random seeds and 95% confidence intervals—an evaluation methodology that directly addresses the window-level inflation prevalent in prior GRF classification literature. The SENN achieved a subject-level ROC-AUC of 0.916 [0.867, 0.964], sensitivity of 0.913 [0.862, 0.963], F1 score of 0.888 [0.851, 0.925], and Average Precision of 0.942 [0.918, 0.967], with post hoc temperature scaling reducing the Brier score from 0.1667 to 0.1511 and producing well-calibrated diagnostic probabilities suitable for clinical threshold setting.

Comparative evaluation against four deep learning baselines—CNN-Residual, BiLSTM, CNN-LSTM, and CNN-Attention—confirmed that the SENN concept bottleneck imposes no statistically significant reduction in discriminative performance across any reported metric, with all pairwise ROC-AUC confidence intervals overlapping. This result constitutes the central empirical validation of the proposed framework: the interpretability constraints are not a performance trade-off but a structural guarantee that comes at zero accuracy cost. Among all five models, the SENN achieves the highest mean sensitivity (0.913), which is the clinically prioritised metric for a screening application where undetected PD cases carry the greatest clinical consequence.

The primary contribution of this work lies in the intrinsic and biologically grounded interpretability of the learned representations. The SENN architecture automatically discovered 16 orthogonal gait concepts, of which seven carry substantial discriminative power: C1 (push-off power reduction, Δ = 3.132), C10 (balance and lateral stability, Δ = 1.653), C12 (step length variability, Δ = 1.496), C13 (heel-to-toe roll-over disruption, Δ = 1.448), C15 (bilateral cadence asymmetry, Δ = 1.006), C14 (swing phase GRF residual, Δ = 0.880), and C16 (overall gait speed proxy, Δ = 0.413). Each concept was linked to its dominant anatomical sensor region through Pearson correlation analysis, and all seven high-discriminative concepts correspond to established PD gait biomarkers documented in the clinical literature. The right midfoot dominance across nine of the 16 learned concepts independently corroborates the clinical observation that PD gait is characterised by a shift from heel-strike to flat-foot contact with prolonged midfoot loading—a finding that emerged from the data without supervision. Unlike post hoc explanation methods such as SHAP and LIME, which approximate decision boundaries after training, the SENN’s relevance scores θ(X) are structurally identical to the prediction itself, guaranteeing that explanations faithfully represent the model’s reasoning for every individual patient.

The clinical implications of these findings are substantial. The combination of competitive sensitivity, calibrated probabilities, and patient-specific concept-level explanations positions the proposed framework as a clinically auditable diagnostic aid rather than a binary classifier. By exposing the specific biomechanical factors—expressed in terms of recognisable gait phenomena—that drive each individual prediction, the SENN satisfies the transparency and accountability requirements that are prerequisites for AI deployment in regulated medical settings. The subject-level decision threshold τ* = 0.81, tuned on the validation set and supported by the full ROC curve, allows clinicians to adjust the sensitivity-specificity operating point according to local clinical priorities without retraining the model.

The current study has several limitations that should be acknowledged. Most notably, the stratified holdout protocol employed here, while statistically rigorous across five independent seeds, is inherently less conservative than leave-one-subject-out (LOSO) cross-validation, which would utilise the full dataset for both training and evaluation without any fixed holdout partition. The relatively small healthy control group (92 subjects) means that the per-seed test set contains approximately 28 healthy subjects, producing wide confidence intervals on specificity that limit the statistical power of cross-model comparisons. Additionally, the evaluation was conducted on a single public dataset, and the concept-to-biomarker assignments have not yet been prospectively validated against expert neurologist assessments. Finally, the framework currently operates on fixed-length walking segments from structured two-minute tests, which constrains its applicability to free-living monitoring scenarios.

Several directions for future work follow directly from these limitations. LOSO cross-validation is recommended as the evaluation standard for submission to higher-impact clinical venues, as it would provide a fully unbiased performance estimate and eliminate the seed-dependent variability observed in specificity across the current runs. Replication on larger independent cohorts is an essential next step before clinical deployment. Incorporating explicit concept supervision—where clinical experts label a small set of reference windows with known biomarker states—could strengthen the biological alignment of the learned representations and reduce the interpretive ambiguity of weakly correlated concepts. Extending the architecture to handle variable-length continuous recordings from free-living instrumented insoles would substantially increase ecological validity and enable longitudinal monitoring of disease progression. Finally, the lightweight architecture of the SENN (four residual blocks, 16 concepts, temperature-scaled output) is well-suited to deployment on edge hardware such as instrumented insole processors or smartphone platforms, enabling non-invasive, continuous, and interpretable gait monitoring outside clinical settings—an avenue of considerable translational importance for early PD detection and remote disease management.

## Figures and Tables

**Table 1:** SENN model hyperparameter values.

Parameter	Value	Description
**Concepts (K)**	16	Number of learned gait concepts
**Backbone filters**	32, 64, 128, 128	Conv1D filters per residual block
**Stochastic depth**	0%, 5%, 10%, 15%	Per-block drop probability (linear schedule)
**SpatialDropout**	0.15	Channel-wise dropout in backbone
**Dense dropout**	0.45	Dropout rate in relevance network
**L2 regularisation**	3 × 10^−4^	Weight decay on convolutional kernels
**λ_div**	1 × 10^−3^	Diversity regularisation coefficient
**λ_spar**	2 × 10^−4^	Sparsity regularisation coefficient
**λ_stab**	5 × 10^−4^	Stability regularisation coefficient
**Label smoothing**	0.05	Cross-entropy smoothing factor
**Batch size**	64	Windows per gradient update
**Optimiser**	AdamW	Decoupled weight decay optimiser
**Learning rate α** **_0_**	5 × 10^−4^	Initial learning rate
**Weight decay λ_wd**	2 × 10^−4^	AdamW decoupled weight decay
**Warm-up epochs**	5	Linear LR ramp before cosine decay
**Max epochs**	120	Maximum training iterations
**Early stopping patience**	12	Epochs without smoothed AUC improvement
**Smoothing window**	3	Rolling mean window for early stopping
**Window size**	128	Timesteps per GRF segment (1.28 s)
**Overlap**	50%	Stride between consecutive windows
**Augmentation copies**	2	Augmented copies per training window

**Table 2:** Subject-level performance comparison of SENN against four deep learning baselines (mean [95% CI], 5 seeds, tuned threshold τ*, subject-level evaluation). Bold indicates the best mean value per metric.

Metric	SENN	CNN-Residual	BiLSTM	CNN-LSTM	CNN-Attention
**Subject ROC-AUC**	**0.916** [0.867, 0.964]	0.914 [0.866, 0.962]	0.914 [0.892, 0.936]	0.909 [0.846, 0.972]	0.926 [0.890, 0.962]
**Sensitivity**	**0.913** [0.862, 0.963]	0.922 [0.874, 0.969]	0.906 [0.840, 0.972]	0.909 [0.835, 0.984]	0.872 [0.806, 0.938]
**Specificity**	**0.671** [0.485, 0.858]	0.586 [0.259, 0.913]	0.700 [0.641, 0.760]	0.636 [0.540, 0.732]	0.764 [0.488, 1.041]
**F1 (PD)**	**0.888** [0.851, 0.925]	0.878 [0.829, 0.928]	0.889 [0.852, 0.926]	0.879 [0.825, 0.932]	0.884 [0.859, 0.908]
**Accuracy**	0.839 [0.782, 0.896]	0.820 [0.733, 0.906]	**0.844** [0.796, 0.891]	0.826 [0.754, 0.898]	0.839 [0.794, 0.884]
**Segment ROC-AUC**	0.885 [0.838, 0.933]	0.885 [0.832, 0.938]	0.885 [0.853, 0.917]	0.882 [0.809, 0.954]	**0.898** [0.852, 0.944]
**Avg Precision**	0.942 [0.918, 0.967]	**0.944** [0.919, 0.969]	0.939 [0.918, 0.960]	0.936 [0.896, 0.975]	0.947 [0.921, 0.973]
**Brier Score ↓**	0.137 [0.106, 0.168]	0.128 [0.094, 0.163]	0.135 [0.110, 0.160]	0.136 [0.090, 0.183]	**0.125** [0.095, 0.156]

Values are mean [95% CI] across 5 independent random seeds (t-distribution, df = 4). Bold = best mean value per row. ↓ = lower is better. CNN-Attention specificity CI exceeds 1.0 due to small healthy test group (n ≈ 28). All models evaluated at tuned threshold τ* on subject-level held-out test set.

**Table 3:** Ablation study results—subject-level performance of SENN under component removal (mean [95% CI], 5 seeds, t-distribution df = 4, tuned threshold τ*). Bold = best mean per metric. ↓ = lower is better.

Condition	Subject-Level Metrics (Mean [95% CI])						
	ROC-AUC	Sensitivity	Specificity	F1 (PD)	Accuracy	Avg Precision	Brier ↓
**► Full SENN (all components)**	**0.916 [0.867, 0.964]**	**0.913 [0.862, 0.963]**	0.671 [0.485, 0.858]	0.888 [0.851, 0.925]	0.839 [0.782, 0.896]	0.942 [0.918, 0.967]	**0.137 [0.106, 0.168]**
**No Diversity Reg (λ_div = 0)**	0.903 [0.857, 0.949]	0.915 [0.863, 0.967]	0.664 [0.533, 0.796]	0.886 [0.852, 0.920]	0.837 [0.793, 0.880]	0.955 [0.932, 0.978]	0.147 [0.113, 0.181]
**No Sparsity Reg (λ_spar = 0)**	0.916 [0.854, 0.979]	0.927 [0.866, 0.988]	0.621 [0.425, 0.818]	**0.893 [0.848, 0.939]**	**0.841 [0.776, 0.906]**	0.958 [0.929, 0.987]	0.144 [0.097, 0.191]
**No Stability Reg (λ_stab = 0)**	0.908 [0.851, 0.965]	0.913 [0.859, 0.967]	0.643 [0.469, 0.817]	0.886 [0.845, 0.926]	0.837 [0.779, 0.894]	0.957 [0.933, 0.981]	0.145 [0.112, 0.178]
**No Stochastic Depth**	0.906 [0.855, 0.958]	0.860 [0.790, 0.929]	**0.743 [0.609, 0.876]**	0.879 [0.841, 0.917]	0.837 [0.790, 0.883]	0.958 [0.934, 0.981]	0.143 [0.108, 0.178]
**No Temp Scaling (T = 1.0)**	0.917 [0.867, 0.968]	0.917 [0.863, 0.972]	0.664 [0.478, 0.851]	0.892 [0.851, 0.933]	0.843 [0.784, 0.903]	**0.962 [0.939, 0.984]**	0.153 [0.122, 0.184]

► Reference condition. All metrics computed on subject-level held-out test set at tuned threshold τ* (maximising macro-averaged F1 on validation subjects). Brier score computed at segment level on calibrated probabilities. 95% CI computed via t-distribution (df = 4). Bold = best mean per column.

## Data Availability

You can access the PhysioNet GRF dataset using the following link: https://physionet.org/content/gaitpdb/1.0.0/ (accessed on 3 January 2026).
